# Effects of mirror therapy preceding augmented reality in stroke rehabilitation: a randomized controlled trial

**DOI:** 10.1186/s12984-025-01820-8

**Published:** 2025-12-24

**Authors:** Chia-Jung Lin, Keh-chung Lin, Hiu-Ying Lau, Yu-wei Hsieh, Yi-chun Li, Wen-Shiang Chen, Chia-Ling Chen, Ya-Ju Chang, Ya-Yun Lee, Grace Yao, Yi-shiung Hrong, Hsiao-Chieh Pan, Yi-Hsuan Wu, Wan-Ling Hsu, Chih-chieh Kuo, Han-ting Tsai, Chih-yu Lin, Pin-chen Chang

**Affiliations:** 1 School of Occupational Therapy, College of Medicine, National Taiwan University, Taipei, Taiwan; 2 Division of Occupational Therapy, Department of Physical Medicine and Rehabilitation, National Taiwan University Hospital, Taipei, Taiwan; 3 Department of Occupational Therapy, College of Medical Science and Technology, Chung Shan Medical University, Taichung, Taiwan; 4 Department of Occupational Therapy and Graduate Institute of Behavioral Sciences, College of Medicine, Chang Gung University, Taoyuan, Taiwan; 5 Department of Occupational Therapy, I-Shou University, Yanchao District, Kaohsiung, Taiwan; 6 Department of Physical Medicine and Rehabilitation, National Taiwan University Hospital, National Taiwan University College of Medicine, Taipei,, Taiwan; 7 Department of Physical Medicine and Rehabilitation, Chang Gung Memorial Hospital, Linkou, Taoyuan, Taiwan; 8 Graduate Institute of Early Intervention, College of Medicine, Chang Gung University, Taoyuan, Taiwan; 9 School of Physical Therapy and Graduate Institute of Rehabilitation Science, College of Medicine, Chang Gung University, Taoyuan, Taiwan; 10 Healthy Aging Research Center, Chang Gung University, Taoyuan, Taiwan; 11 Neuroscience Research Center, Chang Gung Memorial Hospital, Linkou, Taoyuan, Taiwan; 12 School and Graduate Institute of Physical Therapy, College of Medicine, National Taiwan University, Taipei, Taiwan; 13 Department of Psychology, National Taiwan University, Taipei, Taiwan; 14 School of Medicine, Tzu Chi University, Hualien, Taiwan; 15 Department of Physical Medicine and Rehabilitation, Taipei Tzuchi Hospital, Buddhist Tzuchi Medical Foundation, New Taipei City, Taiwan; 16 Department of Physical Medicine and Rehabilitation, Ministry of Health and Welfare, Taipei Hospital, New Taipei City, Taiwan; 17 Rehabilitation Department, Feng Yuan Hospital, Ministry of Health and Welfare, Taichung, Taiwan; 18 Department of Physical Medicine and Rehabilitation, Wan Fang Hospital, Taipei Medical University, Taipei, Taiwan

**Keywords:** Stroke, Mirror therapy, Augmented reality, Gamification, Combinatory regimen

## Abstract

**Background:**

Mirror therapy (MT) and augmented reality (AR) are gaining popularity in stroke rehabilitation. MT uses mirror visual feedback to promote bilateral brain coupling and increase primary motor cortex excitability. AR offers an interactive context of practice for promoting motor and cognitive recovery. MT and AR may complement each other for hybrid interventions in stroke rehabilitation. This study investigated the benefits of MT-primed AR (MT + AR) versus AR group, relative to conventional therapy (CT) for individuals with stroke.

**Method:**

The study randomly assigned 45 stroke survivors to the MT + AR group, the AR, or the CT group, and 44 of them completed the experiment and were included in the analysis. Each treatment session was 90 min, 3 times a week, for 6 weeks. All assessments were administered before, immediately after treatment, and at 3 months. Primary outcome measures were the Fugl-Meyer Assessment-Upper Extremity (FMA-UE) and the Berg Balance Scale (BBS). Secondary outcome measures were the revised Nottingham Sensory Assessment (rNSA), Chedoke Arm and Hand Activity Inventory (CAHAI), Motor Activity Log (MAL), and Stroke Impact Scale Version 3.0 (SIS). Adverse events were monitored before and after each session.

**Results:**

After 6 weeks of treatment, the three groups demonstrated significant improvements in the FMA-UE, BBS, CAHAI, MAL, and SIS. In the between-group comparisons, MT + AR and AR groups demonstrated significant advantages in the BBS, proprioception scale of rNSA and SIS, compared with the CT group. Only the MT + AR group, not the AR group, showed significantly better improvements in the FMA-UE and tactile scale of rNSA than the CT group. The MT + AR and AR alone showed differential benefits in the FMA-UE, tactile scale of rNSA, and SIS; the MT + AR rendered significantly better benefits. There were no significant differences among the three groups in the stereognosis scale of rNSA and MAL. No adverse effects were observed.

**Conclusion:**

MT + AR and AR both effectively enhanced sensorimotor functions, balance and postural control, task performance, and life quality in patients with stroke with moderate-to-severe motor impairments. The results showed that MT + AR and AR were more beneficial for improving stroke survivors’ balance, functional mobility, proprioception recovery, and quality of life than the CT group. Furthermore, the MT + AR revealed better outcomes in the upper limb motor function and tactile sensory recovery. Between the MT + AR and AR comparisons, the MT + AR was more beneficial for improving upper limb motor function, tactile sensory recovery, and quality of life.

*Trial registration* NCT05993091.

## Introduction

Neuro-physical dysfunctions are common sequela after stroke and lead to prolonged impairments [1]. Among various interventions for stroke rehabilitation, mirror therapy (MT) is a cost-efficient, convenient, and widely used neurorehabilitation technique [2]. MT was originally developed in 1997 to treat phantom limb pain and later adapted for stroke rehabilitation [3–5]. Meta-analyses suggest that MT may improve stroke survivors’ sensorimotor impairment, motor function, and activities of daily living [6–9].

The key factor that may contribute to the therapeutic effectiveness of MT for stroke patients is mirror visual feedback. When executing MT, stroke survivors look at the mirror reflection of the less-affected arm, which can help exert a robust influence on the activation of neural substrates, including the primary motor cortex, also known as M1, primary somatosensory cortex, precuneus, and cerebellum [10, 12]. Other proposed mechanisms include perceptual-motor processes and activation of the mirror neuron system [13]. These hypotheses may play a crucial role in contributing to motor relearning and facilitating recovery from physical dysfunction after stroke [12]. The exact processes driving the effectiveness of MT are not yet fully understood.

MT can be delivered in two modes: unilateral MT (UMT), where the affected limb remains static, and bilateral MT (BMT), where the affected limb mirrors the movement of the unaffected limb [3, 14, 15]. These two modes are distinguished by the role of the unaffected limb in the therapeutic process. Several studies have suggested that each mode may offer unique advantages in stroke rehabilitation [13, 14, 16–18]. Additionally, the MT intervention content incorporates both movement-oriented and task-oriented practice. Movement-oriented practice without manipulating objects seemed to be more beneficial to improve motor impairment [16, 19], whereas task-oriented practice may induce greater neural activities in the motor cortex [16]. Therefore, combining BMT and UMT with two practice types can potentially become an optimal MT regimen for stroke rehabilitation.

MT could be a promising priming technique for preparing motor cortex’s excitability, and promoting neuroplasticity, leading to improved clinical outcomes and stroke recovery [20, 21]. Instead of applying a single therapy, previous research has highlighted the effectiveness of hybrid therapy, which involves the sequential or concurrent combination of two or more therapies [20]. Hybrid approaches may complement the benefits of each therapy or enhance overall effectiveness.

Priming helps rebalance and optimize both sides of motor cortex excitability and represents an effective therapeutic approach [20, 22–24]. Among these priming techniques, bilateral priming is a purposeful strategy to pre-activate an individual’s damaged primary motor cortex, rebalancing the excitability of M1, and enhancing motor relearning effects [21]. MT with bilateral practice and multiple sensory inputs holds promise to be an effective priming technique [22]. Numerous MT-related studies have provided supportive evidence for both neural changes that demonstrate neural modulation and long-term neural plasticity [18, 25] and for behavioral changes among stroke survivors [26–28]. To broaden the scope of current research, this study explored the MT priming effect and interactive technology-based rehabilitation.

Augmented reality (AR) is an emerging, cost-effective technology increasingly used in stroke rehabilitation. AR combines real-life images captured by a depth sensor with computer-generated virtual elements, creating an integrated visual experience. The computer-generated images are then projected, providing users with real-time visual feedback and enhancing their interaction with both physical and virtual environments [29–31]. Several studies have applied this technology to stroke clinical trials [29, 31, 32]. Additionally, AR is characterized by its partial user engagement in the simulated environment, which results in less dizziness compared with virtual reality, while integrating real-world elements into the virtual interface [29, 32, 34]. This interactive technology has been recognized as a novel intervention in stroke rehabilitation.

There are advantages of AR-based system in stroke rehabilitation, including gamified entertainment and increasing motivation, movement repetition and duration, providing structured movement practice with a visual cue, and offering individualized context-specific rehabilitation [30–32, 35]. AR has been investigated for its feasibility, effectiveness, and application in stroke rehabilitation. Evidence from several studies has demonstrated that AR may enhance stroke survivors’ balance, gait, lower limb muscle strength and postural control, and decreases fear of falls [29–32]. Furthermore, studies also showed improvement in upper limb recovery, a decrease in functional performance limitation, and decrease activities of daily living dependency [30–32]. Despite the promise of AR for stroke rehabilitation, AR may lack context-specific practice for generalizing learned skills into daily living activities [30].

MT provides task-based practice, including occupational-related practice, which can complement AR and has the potential for motor learning, serving as a promising sensorimotor priming strategy. AR provides gamified, task-oriented interventions that are engaging, motivating, and tailored, thereby integrating essential skills relevant to daily living activities. Combining MT and AR is expected to synergistically enhance rehabilitation outcomes. Despite the growing interest in AR in rehabilitation, no studies to date have specifically examined the effects of AR with and without the priming technique of MT in stroke rehabilitation. Given this gap in the literature, this study was designed to compare the effectiveness of these interventions. The null hypothesis posits that there will be no significant difference in treatment outcomes between the three groups (i.e., MT + AR, AR, and CT) after therapy.

## Methods

### Trial design

**Fig. 1:**
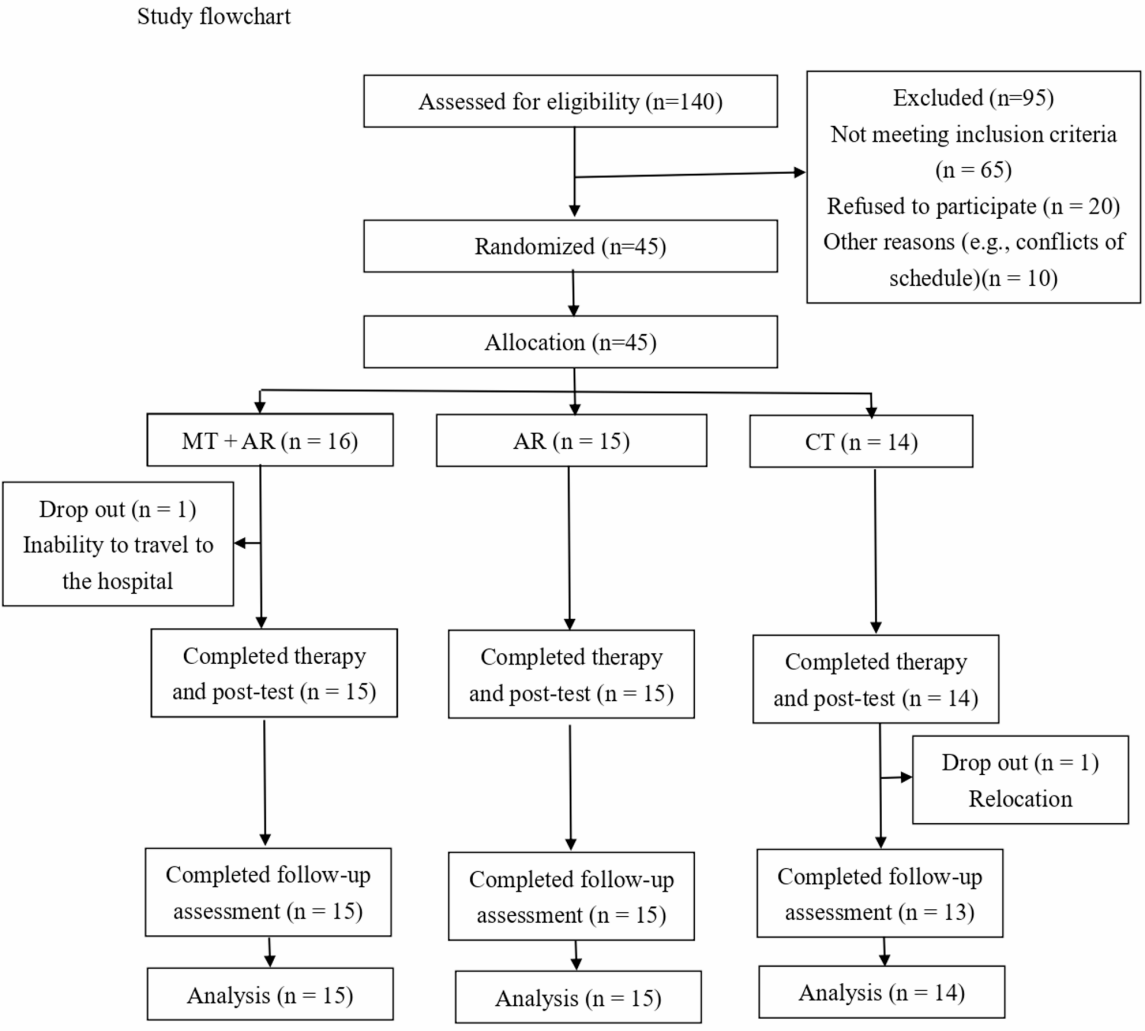
Study flowchart

This study was a three-arm, parallel, randomized-controlled trial with a 3-month follow-up. The study protocol was approved by the institutional review boards of the four recruiting sites and registered at ClinicalTrials.gov (ID: NCT05993091). All participants gave written informed consent. The authors followed the CONSORT 2010 checklist for reporting randomized controlled trials [33]. The Data and Safety Monitoring Board of this study oversaw adverse events and ensured protocol compliance. Seven research personnel involved in the study were trained to administer group randomization, assessments, or treatment delivery. Under the supervision of the principal investigator and senior research personnel, the trained personnel conducted the interventions. All interventionists underwent standardized training before the study and participated in bi-weekly meetings to ensure ongoing monitoring and consistency. Figure 1 shows the study flowchart.

### Participants

The participants were recruited from May 2023 to July 2024. The assignment sequence was generated using an online random number table (accessible at http://www.randomizer.org/). Research personnel, who were not involved in the intervention, conducted the sequence assignment and assessed outcome measurements. After signing informed consents, participants were stratified into four strata based on the side of stroke lesion (right or left hemisphere cerebrovascular lesions) and the initial level of motor impairment, using a cutoff point of 33 in the Fugl-Meyer Assessment-Upper Extremity (FMA-UE) [36]. Participants were randomly assigned to each group in the same ratio.

The inclusion criteria were (1) ≥ 3 months after ischemic or hemorrhagic stroke, (2) age 30 to 80 years old [37], (3) baseline FMA-UE >10 scores [27], (4) Modified Ashworth Scale < 3 [27], (5) Montreal Cognitive Assessment score (MoCA) >24 [38], (6) ability to maintain a step-standing position for ≥ 30 seconds [39], (7) ability to walk ≥ 10 meters independently [40], (8) no severe vision impairments (based on the best gaze subscale score in the National Institutes of Health Stroke Scale) [27], (9) no participation in other studies until the 3-month follow-up, and (10) willingness to provide informed written consent. All participants were able to follow instructions during assessments and treatment.

Exclusion criteria were (1) acute inflammation in the joints of the upper and lower extremities or pneumonia and urinary tract infections [11], (2) receiving botulinum toxin injections within the past 3 months [13], and (3) serious physical or medical comorbidity, including history of epilepsy, that may affect study participation. To determine whether the patient has acute inflammation, study personnel would review the medical chart, conduct clinical observation, and consult the referring physician if needed.

### Interventions

**Fig. 2:**
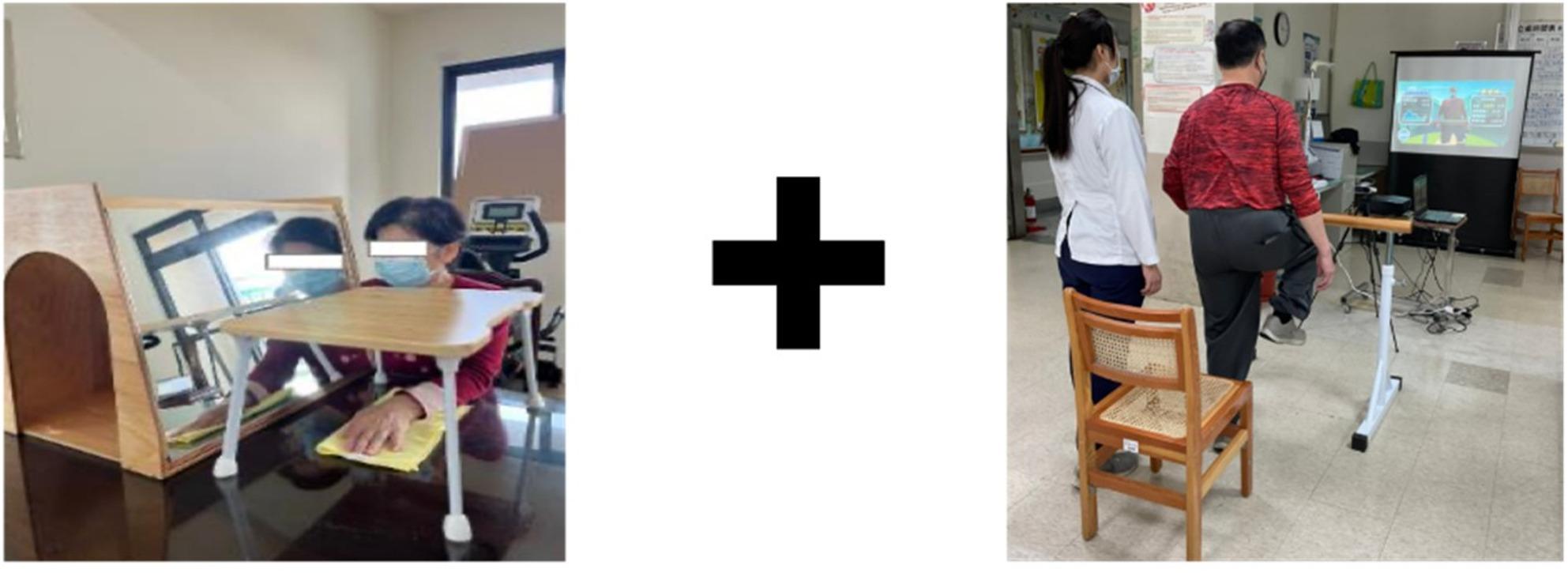
Mirror therapy (left) and augmented reality (right) practice

All participants underwent 90-minute interventions, 3 days per week, for 6 weeks. MT + AR sessions included 40 minutes of MT, 40 minutes of AR practice, and 10 minutes of functional practice (Fig. 2). AR sessions consisted of 80 minutes of AR practice, and 10 minutes of functional practice, whereas CT sessions comprised 90 minutes of conventional occupational therapy, including 10 minutes of functional practice. The goal of functional practice is to generalize the acquired skills to functional activities [27]. Examples of functional practice include peeling fruits, making a cup of tea, and washing dishes.

Based on each participant’s personal needs and rehabilitation goals, the researchers would tailor interventions. Moreover, participants’ impairment levels and relevant factors such as age and baseline physical fitness would be considered to ensure appropriateness and challenge. During the study period, all participants could maintain their routine rehabilitation such as physical therapy or speech therapy that did not focus on bilateral arm training.

In addition to clinical interventions, all participants were provided with a home-based program relative to daily functional activities with the affected upper limb or both upper extremities for 30 minutes per day, 5 days per week. Every participant was provided written instructions, a document log, and home practice tools, such as bottles, towels, and cups. A behavioral contract was signed before the first intervention to promote adherence. Home practice sessions were recorded in the log, including three home practice sessions selected during clinic intervention and six functional practice sessions selected with participants. For adherence monitoring, participants were instructed to bring their recorded sheets at least once a week, or recorded home practice videos, if necessary, to the clinic for review. To maintain treatment outcomes, participants continued the home practice during the 3-month follow-up period. Monthly follow-up calls were conducted to check adherence and adjust program difficulty when needed.

#### MT protocol

At the beginning of MT practice, participants practiced movements without a mirror to ensure task familiarity. Then, a wooden mirror box measuring 41 × 50 × 30 cm was positioned along the participant’s mid-sagittal plane. During the MT practice, a tray table was used to prevent the participant from seeing the unaffected arm [41, 42]. Blocking vision of the unaffected arm can help increase the excitability of the ipsilateral primary motor cortex of the injured brain [43]. During the MT sessions, participants were instructed to focus on observing the reflected movements of the non-affected limb, while being encouraged to mentally simulate similar movements with the affected arm. All participants underwent UMT and BMT in each section. The allocation of UMT and BMT was approximately equal. However, the ratio was adjusted based on the severity of the participant’s motor impairment and their progress throughout the intervention.

**Fig. 3:**
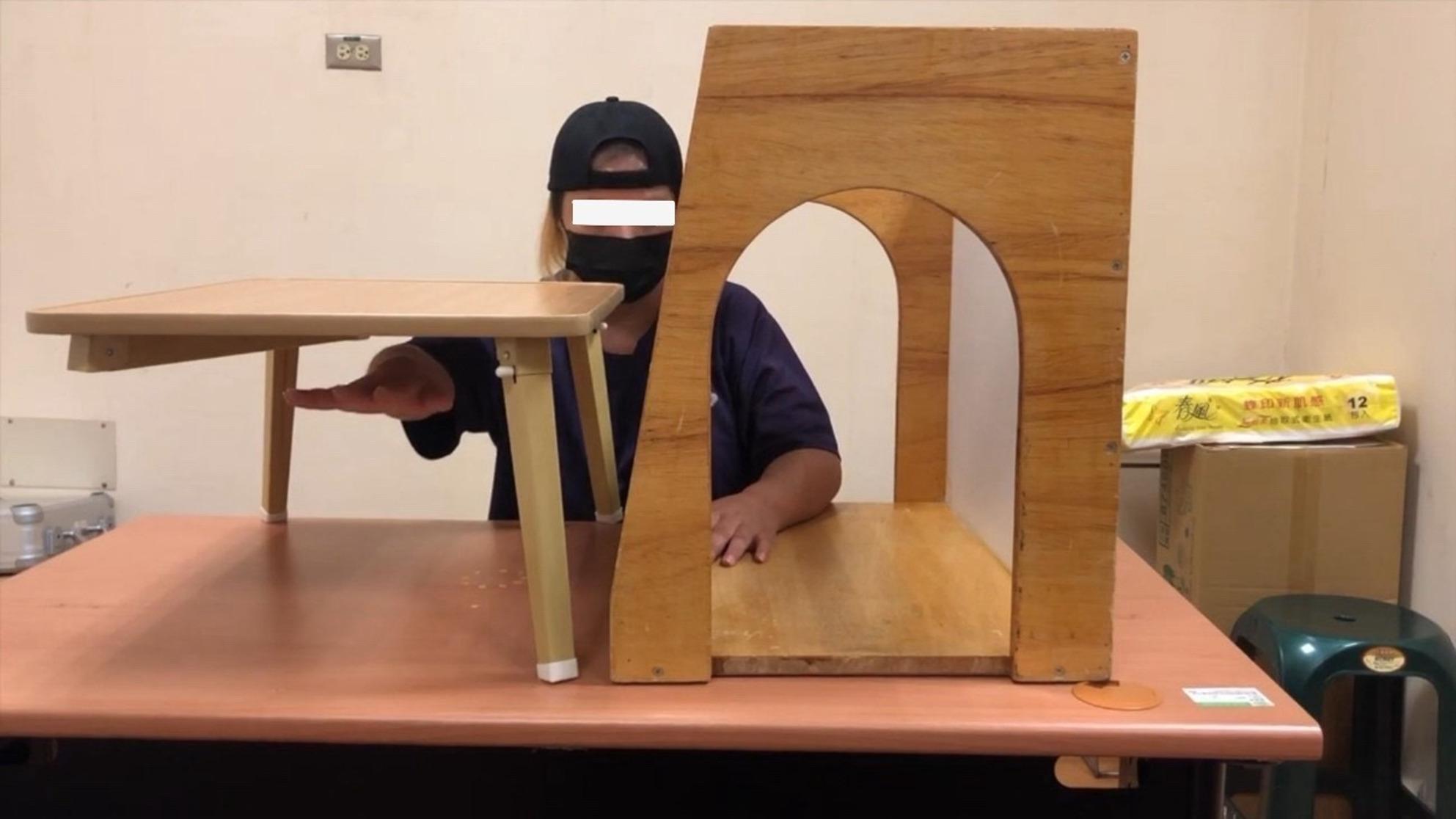
Unilateral mirror therapy with movement-based practice

**Fig. 4:**
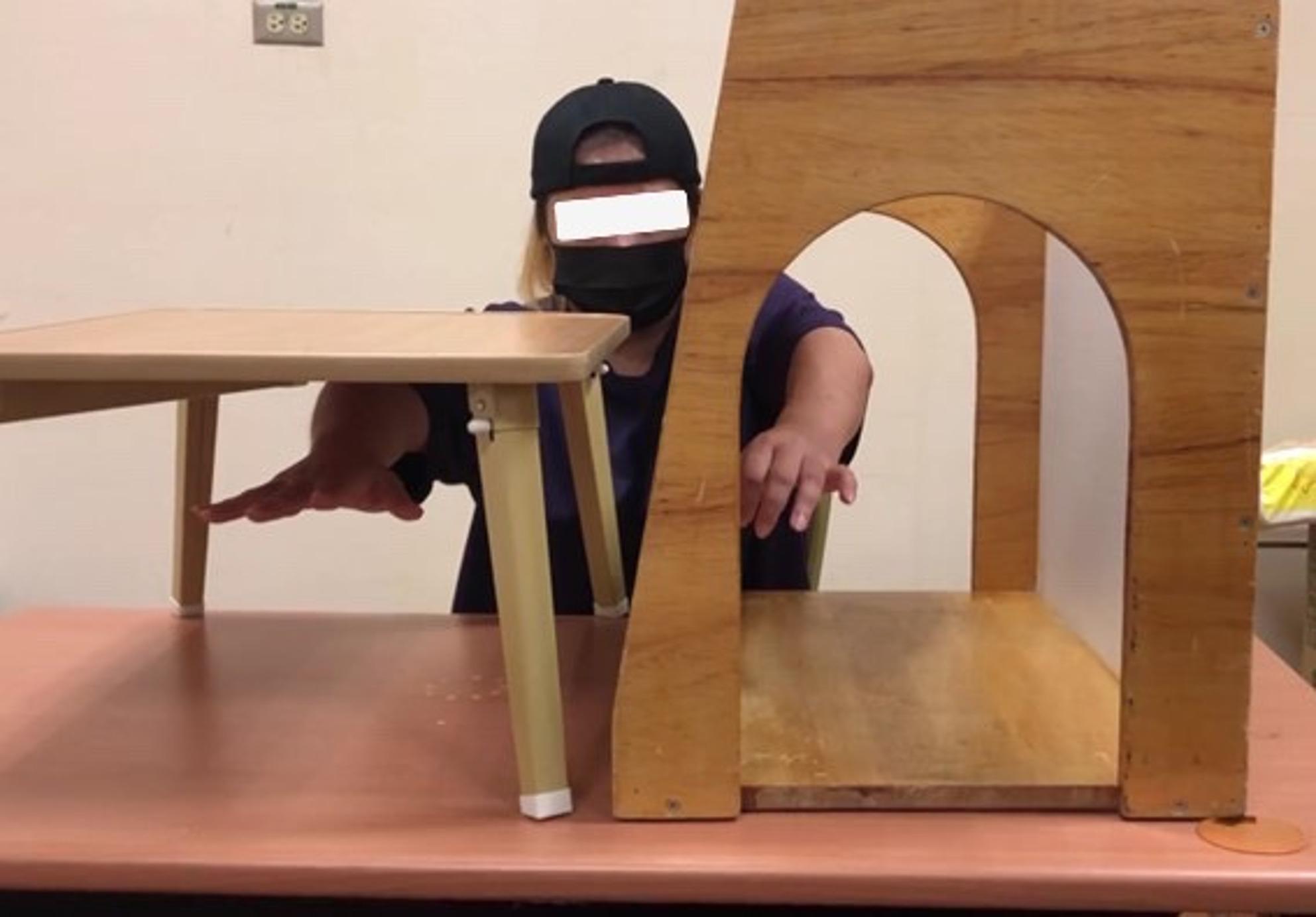
Bilateral mirror therapy with movement-based practice

**Fig. 5:**
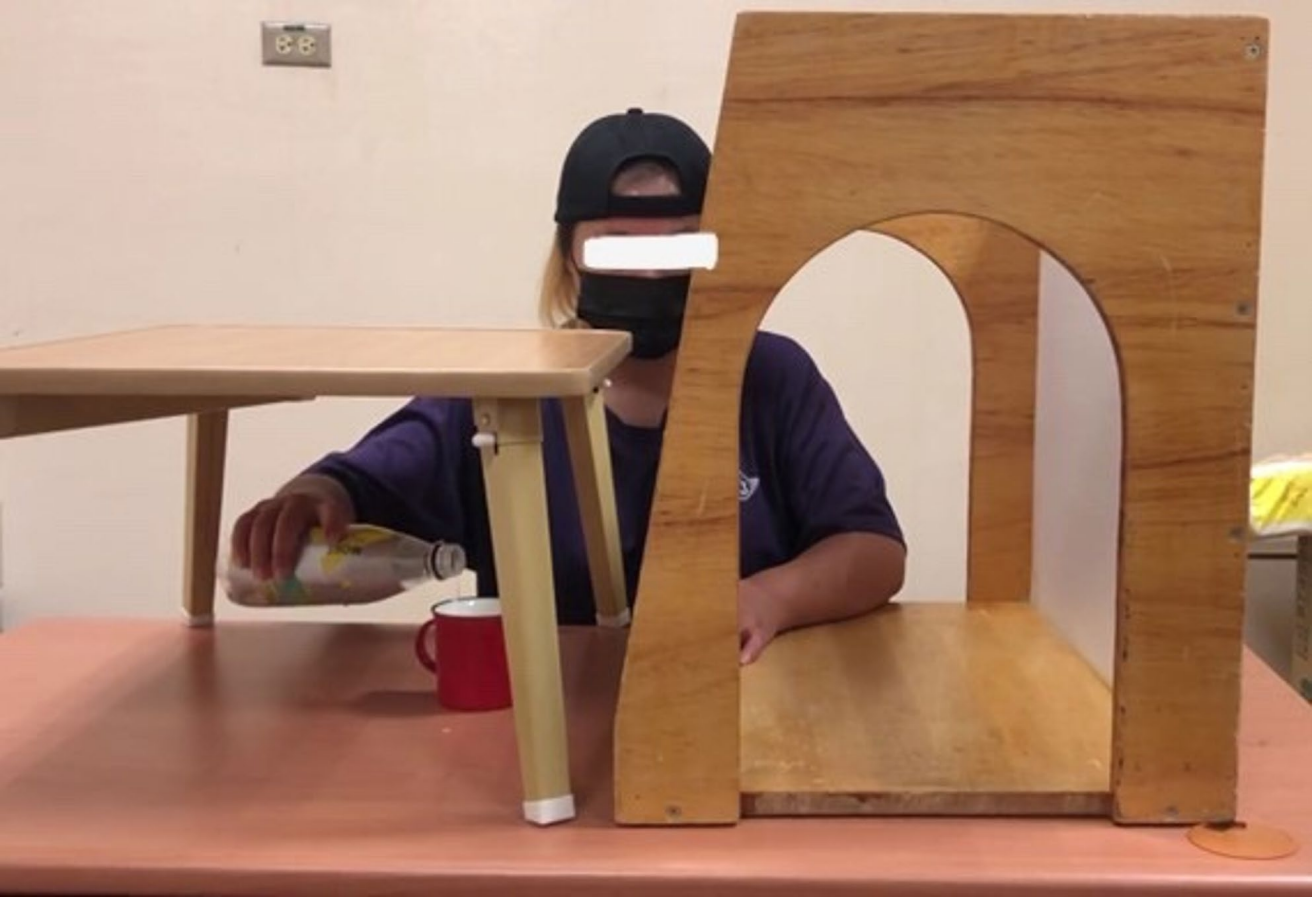
Unilateral mirror therapy with task-oriented practice

**Fig. 6:**
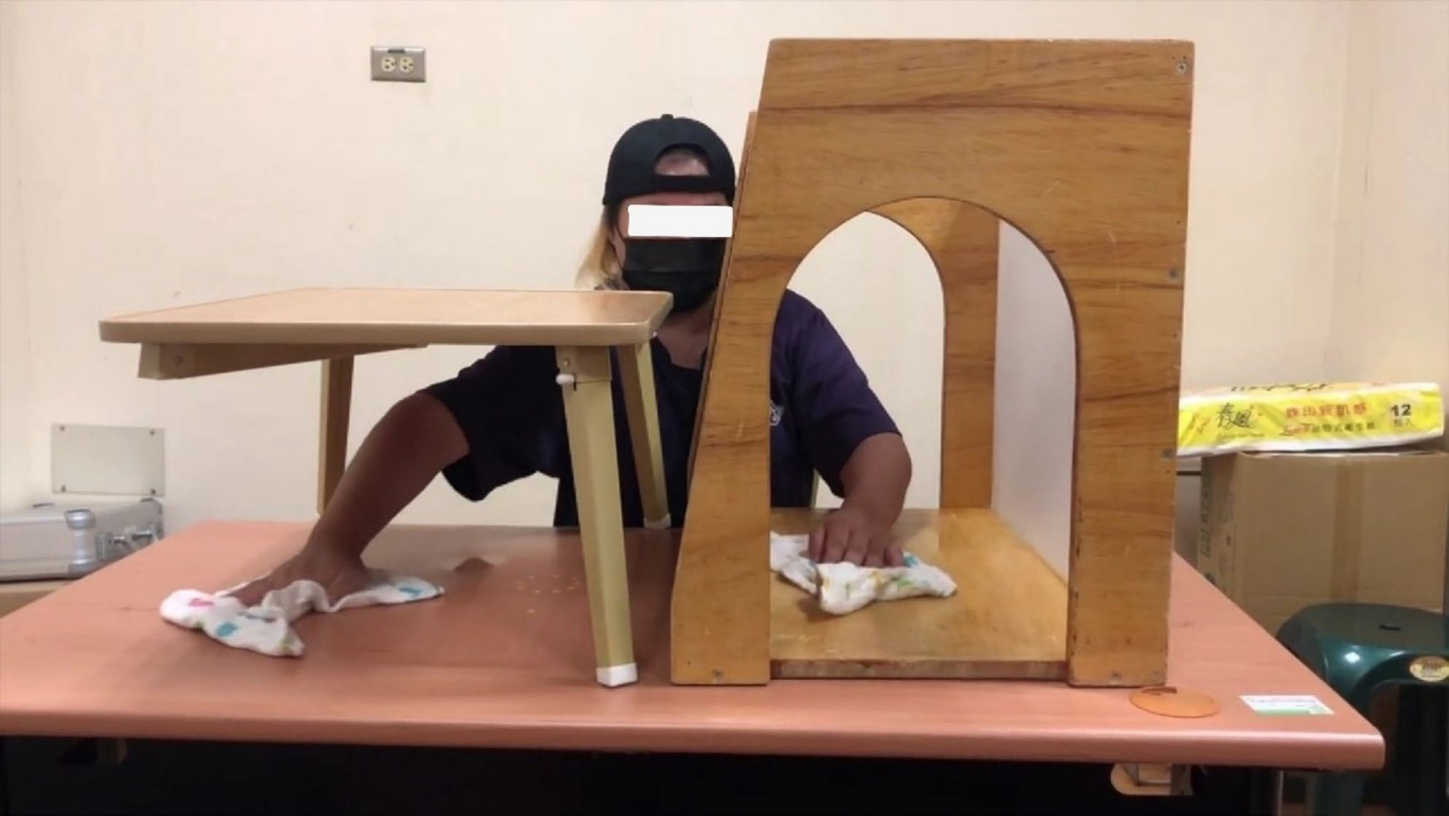
Bilateral mirror therapy with task-oriented practice

The MT activities included gross motor movements (e.g., shoulder flexion, elbow flexion and extension, etc.), fine motor movements (e.g., opposition, finger flexion and extension, etc.), and object manipulation (e.g., reaching and grasping an orange, flipping cards, releasing cones, etc.). Each task was repeated 20 to 30 times, depending on the participants’ proficiency and the difficulty of the tasks. The difficulty of the MT practice was incrementally increased, starting with simple movements and progressing to more complex tasks, ensuring a “just right” challenge tailored to the participant’s improvement. The protocol was modeled after a previous study [42]. Figures 3, 4, 5 and 6 show UMT and BMT practice with movement-based and task-based conditions.

#### AR protocol

In this clinical study, the MT + AR group and the AR group engaged in four types of AR games: upper limb, lower limb, balance and postural control, and cognition (Augmented Reality Based Motion Sensing Serious Game System, Habitz Medtech Co., LTD, Taiwan). Table 1 provides more detailed descriptions of the AR practices. The participants practiced the four games (upper limb, lower limb, balance and postural control, and cognition) in sequence. However, the AR games might be individually adjusted based on the patient’s condition when necessary. The MT + AR group performed each domain for approximately 10 minutes, while the AR group performed each domain for approximately 20 minutes.

**Table 1:** Domains and contents of the AR program and duration of practice in the MT + AR group and the AR group

Group	Duration of practice	AR domains	Contents of the game
MT + AR	10 minutes	Upper limb	Reaching to grasp (reaching out and grabbing the item and placing it at the designated location) Endurance (raising and holding the affected arm at about shoulder level or higher)
AR	20 minutes		
MT + AR	10 minutes	Lower limb	Sit-to-stand (performing sit-to-stand or stand-to-sit training on seats of different heights created in a virtual environment) Endurance (alternatively flexing the lower extremities to about hip level for a few minutes)
AR	20 minutes		
MT + AR	10 minutes	Balance and postural control	Standing on one leg (stepping and hiking up the hill in the virtual environment) Gait tasks (walking back and forth to climbing a mountain and the game records the number of steps and the duration of the activity) Weight shift (standing without moving the feet and picking fruits with left and right hands)
AR	20 minutes		
MT + AR	10 minutes	Cognitive practice	Memory (memorizing and matching pictures arranged in random order) Picture pairing (pairing the same figures on the screen)
AR	20 minutes		

The level of difficulty was selected based on the participant’s age and severity of motor impairments. To ensure safety, a handrail was placed in front of the participant for additional support. The therapist accompanied the participants throughout the intervention for guidance. Goals were set at the beginning of each AR practice session and were applied to functional activities. For example, in the lower limb practice domain, a goal of 100 steps was set. Upon achieving the goal at the end of the practice session, participants were guided to the hallway outside the practice room to apply the skills they had just practiced. In the next session, the goal might be increased to 110 steps to encourage further progress.

#### CT protocol

The CT interventions were tailored to accommodate participants’ levels of motor deficits and their personal needs, including passive and active range of motion exercises, practice of upper and lower limb tasks, balance activities, and functional practice of activities of daily living. The treatment protocols used various occupational therapy techniques, such as bilateral practice, neurodevelopmental approaches, and functional task practice. To alleviate functional deficits, the intervention included exercises targeting the affected arm, gross and fine motor practices, muscle strengthening exercises, and functional task practices. The therapist collaborated with each participant, considering their occupational roles and their priorities, and then selected appropriate functional tasks, such as packing items, cleaning up a table, and folding clothes, etc.

### Outcome measures

Assessments were conducted at baseline, after the 6-week-intervention, and at the end of the 3-month follow-up period. Blinded research personnel conducted assessments.

#### Primary outcome measures

The FMA-UE is an objective outcome measurement for upper limb motor impairment, assessing motor performance of the proximal upper extremities, wrist, hand, and coordination, with a total of 33 items recorded [44]. The validity, reliability, and responsiveness of FMA-UE have been established in stroke [44, 46].

The Berg Balance Scale (BBS) is one of the most widely used tools to evaluate balance and functional mobility. The instrument includes 14 items to assess the participant’s ability to maintain static and dynamic balance with various functional movements, such as picking up an item from the ground, climbing stairs, or transferring from one place to another [47]. The reliability and validity of BBS for stroke patients have been established [48–50].

#### Secondary outcome measures

The revised Nottingham Sensory Assessment (rNSA) evaluates changes of sensations, including tactile sensation, proprioception, and stereognosis [51]. This assessment tool provides a more comprehensive understanding of sensory impairments in stroke patients. In each test, random assessments are conducted with the visual field obscured, allowing the assessor to more accurately determine the extent of the individual’s sensory impairments. The psychometric properties of rNSA have been investigated in the stroke population [51].

The Chedoke Arm and Hand Activity Inventory (CAHAI) assess bilateral upper limb function in daily activities, including pouring water, opening a jar, or zipping up the zipper. CAHAI consists of 13 items that requires stroke survivors to perform with both hands [52]. CAHAI reliability and validity have been established in stroke [53, 54].

The Motor Activity Log (MAL) is a semi-structured, self-reported assessment tool that evaluates the frequency (referred to as MAL-Amount of Use [AOU]) and the quality (referred to as MAL-Quality of Movement [QOM]) of the use of an affected upper limb. It consists of 30 daily life tasks, most of which require collaboration of both hands to complete, such as turning on the light, lifting items, and using utensils [55, 56]. The MAL has demonstrated reliability and validity in assessing motor activity in stroke patients [55].

The Stroke Impact Scale version 3.0 (SIS) is a patient-reported outcome measure of stroke-specific health-related quality of life [57]. The SIS comprises 59 items that evaluate eight domains, including strength, hand function, mobility, activities of daily living, memory, emotion, social participation, and stroke-specific quality of life. The SIS is a valid, reliable, and well-responsive assessment in stroke patients [57].

#### Other test used in this study

The MoCA is a brief test for identifying cognitive impairment [58]. It focuses on cognitive domains, including visuospatial abilities, executive functions, language, delayed recall, working memory, attention, concentration, and orientation to time and place [59]. The validity, reliability, and responsiveness of the MoCA are satisfied [, ].

### Sample size

No study to date has investigated the effects of MT-primed AR or has compared primed AR with unprimed AR and CT. Therefore, the sample size this project required was estimated from previous studies and our pilot study.

Previous study investigated the effectiveness of MT compared with a control group on upper limb function. The results found that MT showed a medium effect size on upper limb function (Hedges’ *g* = 0.51) [60]. Furthermore, hybrid interventions adopting MT as a priming technique also demonstrated effectiveness on FMA-UE (Cohen’s *d* = 0.35) [27]. Additionally, research has provided supportive evidence of AR compared with controlled interventions in improving upper limb motor function (*f* = 0.39) [32]. The continuing pilot study comparing MT + AR (*n* = 15) and conventional therapy (*n* = 12) also showed significant changes in FMA-UE total scores (*f* = 0.47). On the basis of the findings of published literature [32] and the pilot study, it is reasonable to expect a medium-to-large effect size (*f* = 0.39–0.47), considering a statistical power of 0.80 with a two-sided type I error of 0.05 in an analysis of variance. It was estimated that a total sample size of approximately 48 to 69 was needed for this study based on the G*Power 3.1 software (Heinrich Heine University, Düsseldorf, Germany, 2009).

### Statistical analysis

Statistical analyses were conducted using IBM SPSS 24.0 software (IBM Corp., Armonk, NY, 2022). The normality of data was studied based on the value of skewness (± 1). Baseline variables were compared between groups using one-way analysis of variance (ANOVA) for continuous data [61] and the χ^2^ test for categorical data. For between-group comparisons, differences from baseline to after the intervention and at follow-up were compared using two-way mixed ANOVA. The significance level was set at *p* < 0.05. When the two-way mixed ANOVA indicated a significant difference among groups, post-hoc pairwise comparisons were conducted. Paired *t* tests were used to investigate within-group differences between pre-test and post-test assessments as well as between pre-test and follow-up assessments. Missing data were addressed using the last observation carried forward method [62, 63]. The last observation carried forward was used when participants drop out before completing the follow-up assessment. Additionally, the effect size partial eta squared (η^2^) was calculated to measure the magnitude of treatment effects for each outcome measure. Cohen’s (1988) criteria were adopted to interpret the effect size η^2^, with 0.14 = large effect; 0.06 = moderate effect, and 0.01 = small effect [64].

## Results

### Demographics

Of the 140 stroke survivors screened between May 2023 and July 2024, eligible participants were randomly assigned to one of three groups: MT + AR (*n* = 16), AR (*n* = 15), or CT (*n* = 16). A total of 44 participants completed the study and were included in the data analysis (MT + AR = 15, AR = 15, CT = 14). One MT + AR group participant dropped out because of inability to travel to hospital and one CT group participant relocated during the follow-up period and dropped out. Table 2 summarizes the baseline demographic characteristics and pre-test scores of the primary outcome measures. Differences among the three groups were not statistically significant (*p* > 0.05).

**Table 2:** Demographics and baseline clinical characteristics

Baseline characteristics	MT + AR (*n* = 15)	AR (*n* = 15)	CT (*n* = 14)	*P* Value
Age, year, mean (SD)	58.84 (10.92)	57.93 (11.70)	61.17 (13.26)	0.758 ^a^
Gender, n Male/Female	6/9	9/6	8/6	.497^b^
Side of lesion, n Right/Left	7/8	6/9	9/5	.405^b^
Type of diagnosis, n Ischemic/Hemorrhagic	12/3	10/5	10/4	0.708 ^b^
Months after stroke, mean (SD)	10.47 (6.14)	13.00 (5.82)	13.29 (9.42)	.514^a^
NIHSS, mean (SD)	4.57 (2.41)	5.20 (2.86)	4.00 (1.36)	.386^a^
MoCA, mean (SD)	25.93 (1.28)	26.13 (1.48)	26.57 (1.60)	.488^a^
FMA-UE-total score, mean (SD)	31.00 (9.11)	31.33 (10.56)	30.00 (9.54)	0.930 ^a^
BBS, mean (SD)	40.80 (7.46)	37.07 (6.53)	37.43 (9.96)	.387^a^

MT + AR, mirror therapy-primed augmented reality; AR, augmented reality; CT, conventional therapy; NIHSS, National Institutes of Health Stroke Scale; MoCA, Montreal Cognitive Assessment; FMA-UE: Fugl-Meyer Assessment Upper Extremity; BBS, Berg Balance Scale

^a^Analysis by analysis of variance

^b^Analysis by Chi-squared test

### Outcome measures

**Fig. 7:**
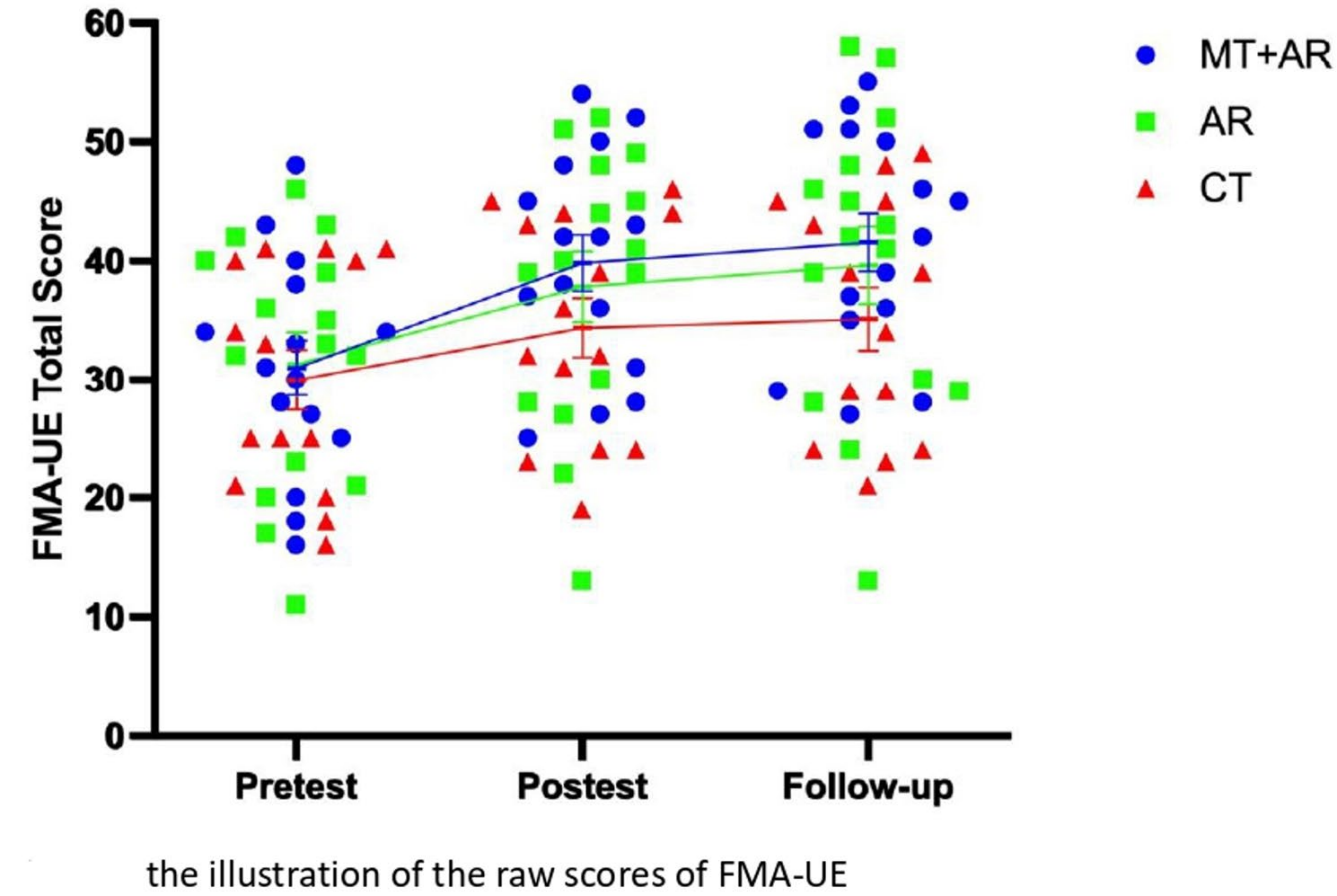
The illustration of the raw scores of the FMA-UE

**Fig. 8:**
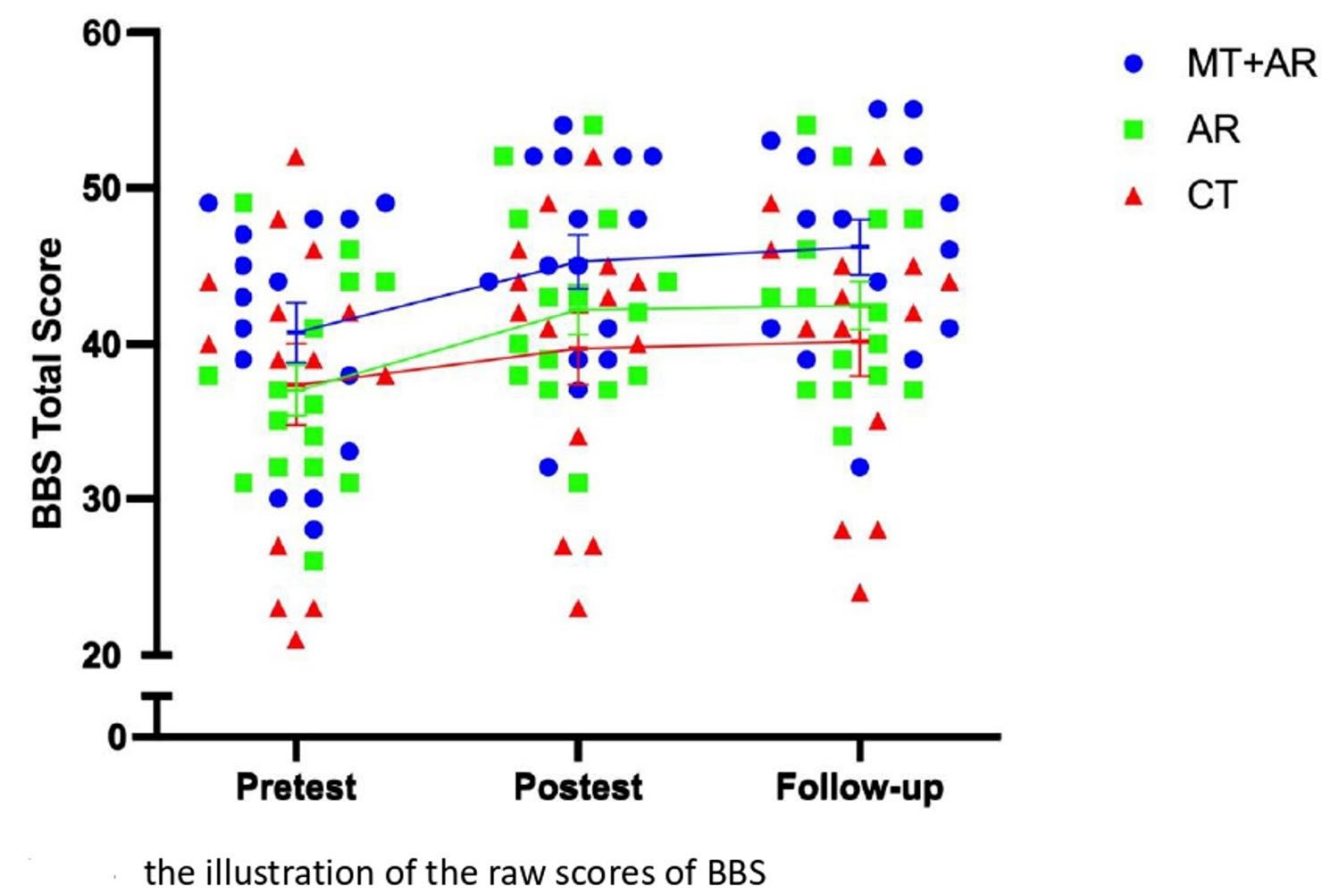
The illustration of the raw scores of the BBS

Tables 3 and 4 present the results of the primary and secondary outcome measures, and Figs. 7 and 8 illustrate the raw scores of FMA-UE and BBS for each treatment group across the three assessment points. Results of post-hoc comparisons indicated that AR + MT showed superior outcomes compared to the CT group in enhancing performance in the FMA-UE, the BBS, the tactile and proprioception scale of the rNSA, and the SIS overall at post-treatment and follow-up assessments. AR without MT was more effective than CT in the BBS and the proprioception scale of the rNSA immediately after intervention and at follow-up. The AR group was superior to CT in the SIS overall at post-test but not at follow-up assessment.

**Table 3:** Descriptive and inferential statistics for primary outcome measures between treatment groups

	Pretest, Mean (SD)			Posttest, Mean (SD)			Follow-up, Mean (SD)			Two-way mixed ANOVA (Group*Time)			Post-hoc comparisons (post-test)		Post-hoc comparisons (follow-up assessment)	
	MT + AR (*n* = 15)	AR (*n* = 15)	CT (*n* = 14)	MT + AR (*n* = 15)	AR (*n* = 15)	CT (*n* = 14)	MT + AR (*n* = 15)	AR (*n* = 15)	CT (*n* = 14)	F	P value	Partial η^2^	Comparison	P value	Comparison	P value
FMA-UE (0–66)	31.00 (9.11)	31.33 (10.56)	30.00 (9.54)	40.40 (10.28)*	37.83 (11.58)*	34.36 (9.40)*	42.1 (10.43)*	39.67 (12.66)*	35.11 (10.09)*	3.27	0.01950	0.15	MT + AR/AR	0.0116 (MT + AR > AR)	MT + AR/AR	0.0288 (MT + AR > AR)
													MT + AR/CT	0.0002 (MT + AR > CT)	MT + AR/CT	0.0002 (MT + AR > CT)
													AR/CT	0.4066	AR/CT	0.2409
BBS (0–56)	40.80 (7.46)	37.07 (6.53)	37.43 (9.96)	45.33 (6.63)*	42.27 (6.19)*	39.79 (8.75)*	46.23 (6.80)*	42.53 (5.96)*	40.21 (8.37)*	4.32	0.0028	0.18	MT + AR/AR	0.7736	MT + AR/AR	0.424040
													MT + AR/CT	0.0003 (MT + AR > CT)	MT + AR/CT	0.0001 (MT + AR > CT)
													AR/CT	0.0001 (AR > CT)	AR/CT	0.0001 (AR > CT)

D, standard deviation, ANOVA, analysis of variance; MT + AR, mirror therapy-primed augmented reality; AR, augmented reality; CT, conventional therapy; FMA-UE: Fugl-Meyer assessment of the upper extremity; BBS, Berg Balance Scale

*p < 0.05 indicates significant within-group difference

**Table 4:** Descriptive and inferential statistics for secondary outcome measures

	Pretest, Mean (SD)			Posttest, Mean (SD)			Follow-up, Mean (SD)			Two-way mixed ANOVA (Group*Time)			Post-hoc comparisons (post-test)		Post-hoc comparisons (follow-up assessment)	
	MT + AR (*n* = 15)	AR (*n* = 15)	CT (*n* = 14)	MT + AR (*n* = 15)	AR (*n* = 15)	CT (*n* = 14)	MT + AR (*n* = 15)	AR (*n* = 15)	CT (*n* = 14)	F	*P* value	Partial η^2^	Comparison	*P* value	Comparison	*P* value
rNSA																
Tactile (0-108)	53.41 (15.38)	52.00 (16.05)	52.35 (12.55)	59.63 (15.07)*	55.33 (15.38)*	53.97 (11.69)*	60.64 (14.63)*	56.00 (15.14)*	54.79 (11.79)*	5.31	0.0007	0.23	MT + AR/AR	0.0012 (MT + AR > AR)	MT + AR/AR	0.0022 (MT + AR > AR)
													MT + AR/CT	0.0001 (MT + AR > CT)	MT + AR/CT	0.0001 (MT + AR > CT)
													AR/CT	0.0578	AR/CT	0.1484
Proprioception (0–21)	10.44 (3.35)	11.00 (3.02)	9.54 (3.15)	13.19 (3.72)*	13.40 (3.76)*	10.32 (2.66)*	13.69 (3.81)*	13.78 (3.80)*	10.75 (2.66)*	3.18	0.0175	0.13	MT + AR/AR	0.6810	MT + AR/AR	0.57162
													MT + AR/CT	0.0014 (MT + AR > CT)	MT + AR/CT	0.0031 (MT + AR > CT)
													AR/CT	0.0059 (AR > CT)	AR/CT	0.0142 (AR > CT)
Stereognosis (0–22)	12.93 (2.87)	12.20 (3.00)	11.21 (3.05)	15.02 (3.62)*	13.56 (3.14)*	12.11 (2.53)*	15.09 (3.25)*	13.78 (3.25)*	12.33 (2.86)*	1.38	0.2470	0.07	N/A	N/A	N/A	N/A
CAHAI (7–91)	43.10 (10.72)	42.15 (14.24)	41.05 (12.43)	50.20 (11.61)*	48.52 (13.33)*	45.72 (12.93)*	51.98 (12.54)*	50.17 (13.20)*	47.77 (13.93)*	2.17	0.0827	0.10	N/A	N/A	N/A	N/A
MAL																
AOU (0–5)	2.49 (0.37)	2.43 (0.46)	2.33 (0.44)	2.93 (0.51)*	2.78 (0.45)*	2.68 (0.58)*	3.10 (0.54)*	2.89 (0.43)*	2.79 (0.54)*	0.38	0.6888	0.02	N/A	N/A	N/A	N/A
QOM (0–5)	2.49 (0.37)	2.41 (0.44)	2.36 (0.43)	2.97 (0.53)*	2.85 (0.51)*	2.63 (0.48)*	3.12 (0.55)*	2.94 (0.45)*	2.77 (0.54)*	0.71	0.5869	0.03	N/A	N/A	N/A	N/A
SIS overall (0-100)	47.83 (12.52)	49.54 (12.81)	47.56 (13.00)	55.52 (11.84)*	56.42 (13.43)*	52.66 (12.24)*	57.36 (11.25)*	59.17 (12.80)*	54.18 (12.83)*	2.82	0.0301	0.12	MT + AR/AR	0.0310 (MT + AR > AR)	MT + AR/AR	0.0620
													MT + AR/CT	0.0002 (MT + AR > CT)	MT + AR/CT	0.00019 (MT + AR > CT)
													AR/CT	0.0410 (AR > CT)	AR/CT	0.0564

SD, standard deviation, ANOVA, analysis of variance; MT + AR, mirror therapy-primed augmented reality; AR, augmented reality; CT, conventional therapy; rNSA, Revised Nottingham Sensation Assessment Scores; CAHAI: Chedoke Arm and Hand Activity Inventory; MAL, Motor Activity Log; AOU: amount of use; QOM: quality of movement; SIS, Stroke Impact Scale; N/A denotes not applicable because of non-significant group*time effects

*p < 0.05 indicates significant within-group difference

MT + AR was significantly more effective than AR alone in the FMA-UE and the tactile scale at post-treatment and follow-up assessments. The MT + AR group was superior in enhancing life quality as rated by the SIS among the three groups. Wilcoxon signed-rank tests revealed that the MT + AR group showed significant improvements on six SIS subscales—strength, hand function, ADL, mobility, memory, and emotion—from pre- to post-intervention. These gains, reflecting enhanced physical and cognitive function, were maintained at follow-up. In contrast, no significant changes were observed in communication and social participation at either time point. No significant difference in the outcome of functional arm use as rated by the MAL among the treatment groups. No adverse response was reported.

## Discussion

### Summary of findings

To the best of our knowledge, this is the first study comparing MT + AR, AR, and CT in stroke survivors with moderate-to-severe motor impairment. The results indicated improvements of sensorimotor, activities of daily living, and quality of life in study participants. The results of this study also rejected the null hypothesis by demonstrating significant between-group differences across the three intervention arms, thereby supporting the efficacy of AR-based interventions compared to conventional therapy. Furthermore, MT + AR showed superior outcomes related to upper limb sensorimotor recovery and quality of life, whereas interventions incorporate with AR were more beneficial in increasing balance and postural control, as well as proprioception.

No serious adverse responses were observed. Some participants experienced transient fatigue or mild limb soreness post-session, which typically resolved after brief rest periods.

### MT-primed hybrid intervention in stroke rehabilitation

The results showed that participants in the hybrid intervention combined with MT had a trend to improve motor impairment compared with the AR and CT groups. These results are similar to those of Rong et al. (2021), suggesting that although all participants significantly improved after the interventions, those who underwent mirror visual feedback before motor practice demonstrated greater motor recovery [28]. Consistent results can also be found in the Ding et al. (2019) study [26]. They reported that a MT-primed intervention showed statistically significant improvement in FMA-UE compared with the control group. However, methodological differences should be noted: prior studies varied in participant criteria, MT dosage, session length, task types, and involvement of follow-up period.

Some possible explanations that may contribute to the motor impairment recovery are as follows. Firstly, task difficulty progressed from simple to complex movements, consistent with protocols in previous studies demonstrating MT’s efficacy in enhancing motor recovery [42]. Second, the MT protocol, which integrates UMT and BMT, demonstrated outcomes consistent with previous studies, indicating its effectiveness in improving motor impairment and function [13, 14, 16]. In the current study, participants in the MT-primed group showed significant improvements likely due to the repetitive movement practice inherent in MT [42]. Furthermore, MT has the potential to enhance self-awareness and spatial attention by stimulating regions such as the superior temporal gyrus, precuneus, and posterior cingulate cortex [65, 66]. The engagement with MT might subsequently recruit the premotor cortex or balance neural activation in the primary motor cortex, specifically towards the affected hemisphere, then aiding in recovery from motor impairment [67, 68]. Lastly, it’s possible that the MT’s augmented visual feedback could be a useful strategy for motor recovery. MT + AR would have a greater advantage than CT, which only relied on intrinsic feedback [16].

Although there were some differences in the motor tasks across the three groups, the MT + AR group performed upper-limb tasks using a mirror box, while the AR group did not include mirror therapy. However, this does not necessarily indicate that the MT + AR group engaged in more upper-limb motor tasks than the other groups. Both the MT + AR and AR groups participated in AR games that likely involved both stability and mobility of the upper limb during task exercises. The CT group, on the other hand, did not include MT or AR but involved a range of tasks such as passive and active range of motion exercises, upper and lower limb exercises, balance activities, and functional practice of daily living tasks, as well as various occupational therapy techniques, including bilateral movement practice, neurodevelopmental approaches, and functional task training. While the MT + AR program differed from the CT program in task modality, it may not have involved a greater volume of upper-limb motor practice. Future research may consider using wearable sensors, such as wrist accelerometers, to provide more precise measurements of upper-limb motor task practice across different treatment groups.

The rNSA findings showed that MT + AR can be more beneficial for tactile sensation improvement among the groups. The observed benefits may be associated with the activation of multimodal neurons. These neurons, situated in the posterior parietal and premotor cortical regions, respond to sensory stimuli such as visual inputs and movement stimuli [69, 70]. The sensory inputs provided by the interventions could modulate the somatosensory cortex network, thereby facilitating the recovery of somatosensation [68, 71]. However, other study found no significant changes in stereognosis post-intervention [72]. Further study is therefore needed to investigate the potential mechanism between MT and the changes in sensory and neuroplasticity in the central neural system.

No significant difference on the enhancement of functional performance between the groups was observed in current study. Yet, the between-group comparison showed marginal significance with a moderate-to-large effect size. The trend is consistent with previous studies [9, 73, 74]. Kim et al. (2016) have suggested that MT may activate the premotor area, a crucial region for motor control and recovery after brain damage [73]. This activation enhances activity in the impaired primary motor area and improves upper limb function. Improving motor function may lead to increased life quality, reflected on the results of SIS [27, 75]. However, the study conducted by Wu et al. [68] reported varying outcomes, indicating that MT did not enhance functional performance in stroke survivors. This was attributed to entrenched living habits that posed challenges for modification.

Post hoc analysis of SIS subscales of the MT + AR group indicated the benefits of the regimen on the subscales of physical function, including strength, hand function, ADL, mobility, as well as memory, and emotion immediately after treatment. The findings indicated the benefits of MT + AR in improving motor and daily functions and aspects of cognitive function, such as memory and picture pairing. The MT + AR group did not show improvements in communication or social participation subscales, as the program did not address language communication or role functions—such as spirituality, altruism, and return-to-work—that are part of the participation domain in the SIS.

### Effects of the AR intervention

The findings of this study supported that AR may be more effective in enhancing balance and postural control among stroke survivors compared to the conventional intervention, which was similar to previous studies [35, 62, 63]. Some possible reasons can be considered. First, AR practice sessions can be conducted in sitting or standing positions, enabling graded task difficulty. Participants who improved could progress from sitting to standing, adding challenge. Second, AR games are standardized, with visual cues that help maintain movement quality. Third, four domains of the AR program consist of balance training, which provide a wide range of balance challenges and further improve the outcomes of BBS [76]. These possible reasons are also mentioned in previous studies, highlighting the beneficial effects of AR-based interventions on lower limb rehabilitation among stroke patients, demonstrating improvements in dynamic balance, gait, and physical performance [29–32, 78].

In addition, the results of this study found both MT + AR and AR significantly improve stroke survivors’ proprioception with a retention until follow-up. Multisensory stimulations provided by AR, including visual, auditory, and proprioceptive feedback may activate pathways of sensory transmission, potentially facilitating therapeutic changes after treatment. AR’s strong proprioceptive feedback may also enhance body position awareness [31], consistent with VR-based proprioception gains reported by Cho et al. (2014) [79]. The posterior column–medial lemniscal pathway, which carries proprioceptive, pressure, and fine touch sensations [80], may underlie the sensory improvements observed, though further research is needed.

Gamified practice activities may increase motivation, potentially enhancing functional improvements and activities of daily living performance [28]. These enhancements could lead to better daily performance and a higher self-perceived quality of life in the SIS. Furthermore, exergaming has been shown to foster positive experiences and a sense of achievement, increasing motivation and emotional well-being [81]. In this study, AR participants achieved superior SIS outcomes compared with CT, supporting AR’s integration into rehabilitation programs to promote recovery and quality of life after stroke.

### Maintenance programs for outcome retention

Many improvements observed at follow-up may be attributed to consistent home practice. High adherence to home protocols can be essential for improvement [13], despite prior research indicating low adherence to stroke rehabilitation protocols [82]. The Dawson et al. (2021) study indicated that incorporating home exercises in addition to clinic therapy can lead to significant improvements in the FMA, even for chronic stroke survivors with moderate-to-severe motor impairment [83].

Monthly contact and the option to share practice videos allowed therapists to support participants and maintain motivation. These strategies encouraged the integration of the affected limb into daily life, reinforcing functional independence. A participant who encountered difficulties with practice execution could contact researchers via communication software contact, and the researchers would then provide instructional videos. This study emphasized the critical role of home practice in promoting recovery and activity for stroke rehabilitation.

### Study limitations

The study has several limitations. Firstly, ten patients with stroke were eligible for inclusion to the study. Some of them were not enrolled due to conflicts of schedule. The total number of study participants was 45, which is close to the lower limit of the sample size needed for the study. The constrained sample size posed challenges in generalizing findings to the broader population and potentially introduced a type II error, reflecting an inability to detect group disparities; that is, the findings may not be generalized beyond the scope of the present study, and the sample size used in the analysis maybe under-powered. Further study is needed to validate the findings with a larger study sample with a longer term of follow-up after treatment.

Second, during the follow-up, conventional therapy outside the study protocol was not standardized, which may have introduced variability. Future studies should standardize or systematically record treatment dosages to better evaluate intervention efficacy.

Third, interventions were partially individualized to align with patient goals, leading to minor variations in content. However, all therapists operated under consistent supervision and attended regular meetings to ensure fidelity.

Lastly, the exclusion criterion in our study may limit the generalizability of the results, as individuals with acute inflammatory conditions or recent Botox injections, a group potentially representative of broader clinical populations, were not included.

## Conclusion

This study aimed to explore the effects of AR, both with and without the priming technique of MT, in comparison to CT. The results of the three dose-matched groups indicated that the MT + AR intervention produced greater improvements than CT in upper limb sensorimotor recovery, balance, and quality of life at both post-treatment and follow-up assessments. The AR intervention alone was more effective than CT in improving balance and proprioception both after the intervention and at follow-up. Additionally, AR alone showed superior effects on overall quality of life at the post-treatment assessment, although this advantage was not maintained at follow-up. Clinicians should consider patient-specific goals when selecting interventions. However, due to the modest sample size, further research with larger cohorts and controlled conditions is warranted.

## Data Availability

The data sets used and/or analyzed during the current study are available from the corresponding author on reasonable request.

## Abbreviations

MT: Mirror therapy
BMT: Bilateral mirror therapy
UMT: Unilateral mirror therapy
AR: Augmented reality
MT + AR: Mirror therapy-primed augmented reality
CT: Conventional therapy
FMA-UE: Fugl-Meyer Assessment-Upper Extremity
BBS: Berg Balance Scale
rNSA: Revised Nottingham Sensation Assessment
CAHAI: Chedoke Arm and Hand Activity Inventory
SIS: Stroke Impact Scale version 3.0
ANOVA: Analysis of variance
MoCA: Montreal Cognitive Assessment
